# Supplementary material for: Partial Hepatic Vein Occlusion and Venous Congestion in Liver Exploration Using a Hyperspectral Camera: A Proposal for Monitoring Intraoperative Liver Perfusion
Source: Cancers (Basel). 2023 Apr 21;15(8):2397. doi: 10.3390/cancers15082397 (PMC10136658; doi:10.3390/cancers15082397)
Supplement: Supplementary file 1 [file cancers-15-02397-s001.zip › cancers-2298232-supplementary.pdf]

SUPPLEMENTARY MATERIALS

Supplementary Figure S1. 3-dimensional plot of the absorbance variation among the spectra between liver lobes. A) t0, B) t1, C) t2, D) t3, E) t4.

A.

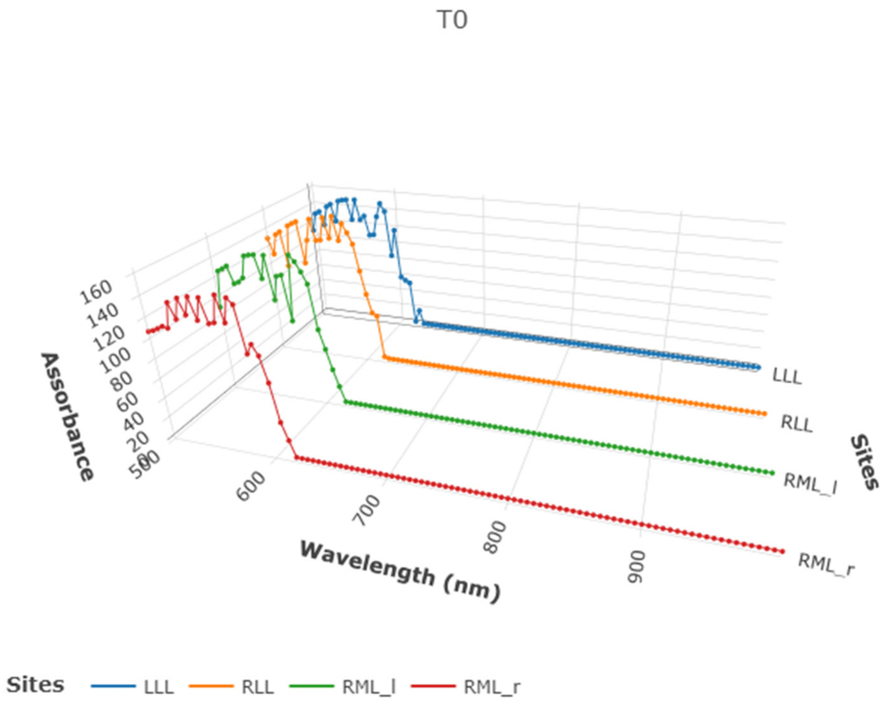

B.

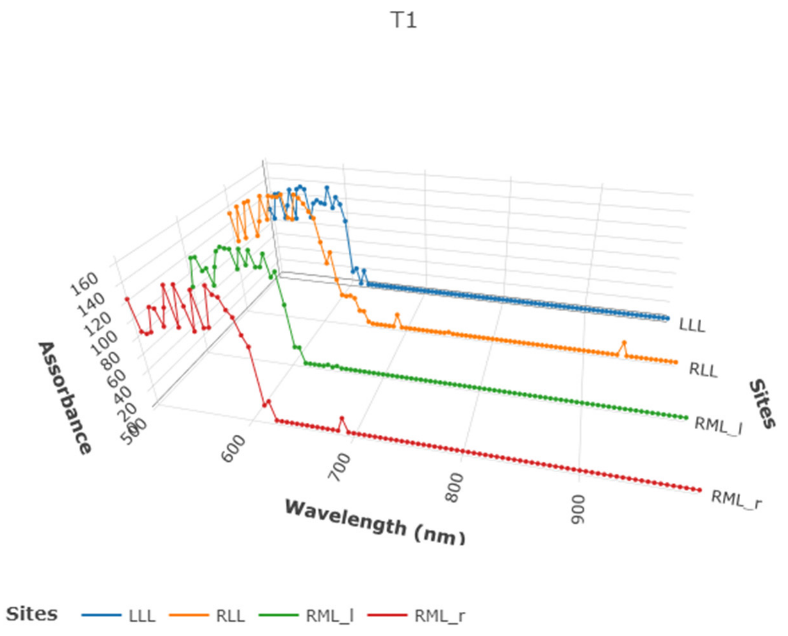

C.

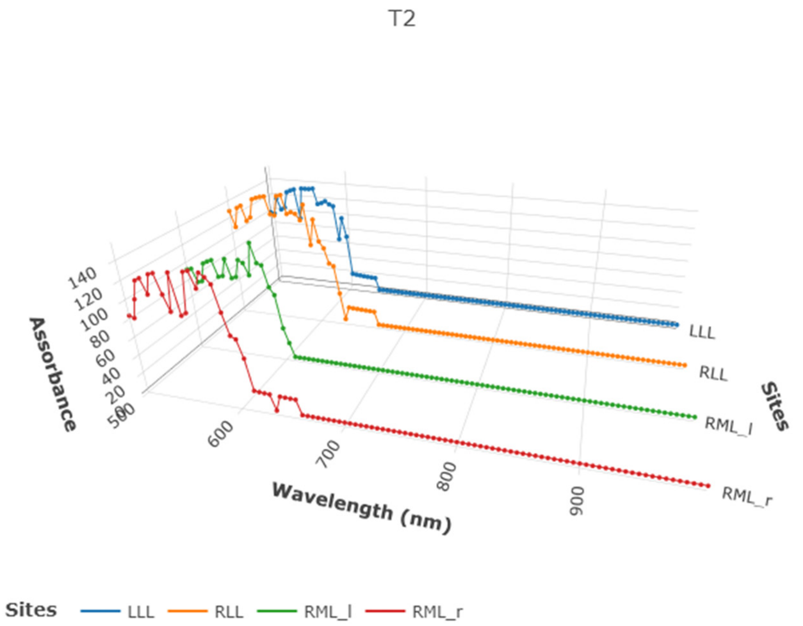
T2
Assorbance
140
120
100
80
60
40
20
0
500
600
700
800
900
Wavelength (nm)
LLL
RLL
RML_l
RML_r
Sites
Sites
LLL
RLL
RML_l
RML_r

D.

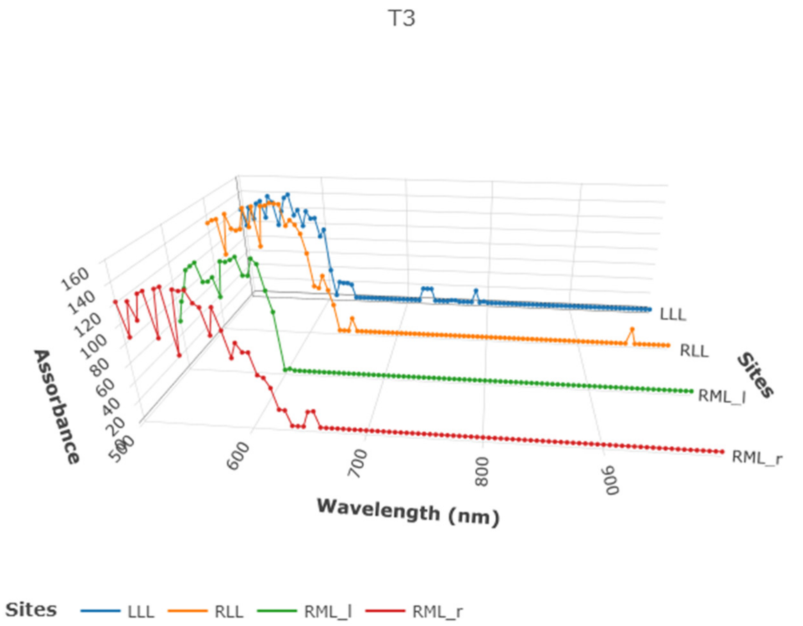
T3
Assorbance
160
140
120
100
80
60
40
20
0
500
600
700
800
900
Wavelength (nm)
LLL
RLL
RML_l
RML_r
Sites
Sites
LLL
RLL
RML_l
RML_r

E.

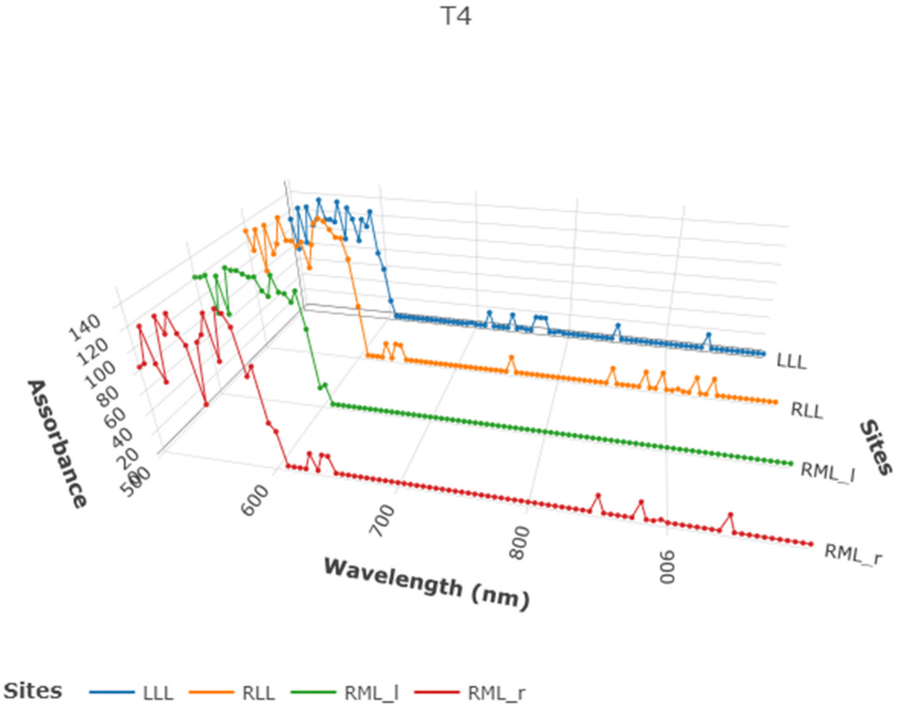
T4
Assorbance
140
120
100
80
60
40
20
0
500
600
700
800
900
Wavelength (nm)
Sites
LLL
RLL
RML_l
RML_r
Sites
LLL
RLL
RML_l
RML_r

Supplementary Table S1. Random effects report of the mixed-effects linear regression to estimate the variations of the hyperspectral indexes among different liver lobes. In this table, the reference level is the left lateral lobe.

T	index		Parameter	Coefficient
0	StO2%	SD	(Intercept: ID)	16.87
		SD	(Residual)	7.17
	NIR	SD	(Intercept: ID)	7.05
		SD	(Residual)	10.4
	THI	SD	(Intercept: ID)	9.31
		SD	(Residual)	7.99
	DeHb	SD	(Intercept: ID)	14.15
		SD	(Residual)	7.76
1	StO2%	SD	(Intercept: ID)	4.02
		SD	(Residual)	7.11
	NIR	SD	(Intercept: ID)	1.33
		SD	(Residual)	14.02
	THI	SD	(Intercept: ID)	11.49
		SD	(Residual)	4.71
	DeHb	SD	(Intercept: ID)	8.6
		SD	(Residual)	4.95
2	StO2%	SD	(Intercept: ID)	14.01
		SD	(Residual)	9.91
	NIR	SD	(Intercept: ID)	20.87
		SD	(Residual)	12.97
	THI	SD	(Intercept: ID)	9.21
		SD	(Residual)	7.66
	DeHb	SD	(Intercept: ID)	11.58
		SD	(Residual)	4.46
3	StO2%	SD	(Intercept: ID)	11.91
		SD	(Residual)	8.45
	NIR	SD	(Intercept: ID)	7.83
		SD	(Residual)	14.22
	THI	SD	(Intercept: ID)	10.07
		SD	(Residual)	6.11
	DeHb	SD	(Intercept: ID)	12.16
		SD	(Residual)	8.21
4	StO2%	SD	(Intercept: ID)	13.98
		SD	(Residual)	7.55
	NIR	SD	(Intercept: ID)	7.83
		SD	(Residual)	4.16
	THI	SD	(Intercept: ID)	9.45
		SD	(Residual)	6.32
	DeHb	SD	(Intercept: ID)	12.43
		SD	(Residual)	6.25

Supplementary Table S2. Random effects report of the mixed-effects linear regression to estimate the variations of the hyperspectral indexes among different liver lobes. In this table, the reference level is the right lateral lobe, after the exclusion of the left lateral lobe.

T	index		Parameter	Coefficient
0	StO2%	SD	(Intercept: ID)	17.87
		SD	(Residual)	7.17
	NIR	SD	(Intercept: ID)	7.59
		SD	(Residual)	12.03
	THI	SD	(Intercept: ID)	9.44
		SD	(Residual)	9.54
	DeHb	SD	(Intercept: ID)	14.87
		SD	(Residual)	8.6
1	StO2%	SD	(Intercept: ID)	3.47
		SD	(Residual)	4.29
	NIR	SD	(Intercept: ID)	8.25
		SD	(Residual)	13.13
	THI	SD	(Intercept: ID)	11.57
		SD	(Residual)	3.74
	DeHb	SD	(Intercept: ID)	7.25
		SD	(Residual)	4.86
2	StO2%	SD	(Intercept: ID)	10.79
		SD	(Residual)	8.91
	NIR	SD	(Intercept: ID)	20.31
		SD	(Residual)	14.55
	THI	SD	(Intercept: ID)	8.58
		SD	(Residual)	7.8
	DeHb	SD	(Intercept: ID)	10.4
		SD	(Residual)	4.23
3	StO2%	SD	(Intercept: ID)	10.28
		SD	(Residual)	9.34
	NIR	SD	(Intercept: ID)	11.09
		SD	(Residual)	13.99
	THI	SD	(Intercept: ID)	9.23
		SD	(Residual)	6.75
	DeHb	SD	(Intercept: ID)	10.94
		SD	(Residual)	7.21
4	StO2%	SD	(Intercept: ID)	11.83
		SD	(Residual)	7.76
	NIR	SD	(Intercept: ID)	7.59
		SD	(Residual)	4.46
	THI	SD	(Intercept: ID)	8.51
		SD	(Residual)	6.87
	DeHb	SD	(Intercept: ID)	11
		SD	(Residual)	6.12

Supplementary Table S3. Mixed-effects linear regression to estimate the variations of the hyperspectral indexes among different liver lobes. In this table, the reference level is the right medial lobe – left side, after the exclusion of the left lateral lobe. Above, the random effects were reported.

T	INDEX	SITE	Coefficient	SE	95% CI	t	df	p
		**(Intercept)**	42.83	7.86	[25.85, 59.81]	5.45	13	< 0.001
	**StO2%**	**RLL**	-4	4.14	[-12.95, 4.95]	-0.97	13	0.352
		**RMLr**	-3	4.14	[-11.95, 5.95]	-0.72	13	0.482
		**(Intercept)**	59.83	5.81	[47.29, 72.38]	10.3	13	< 0.001
	**NIR**	**RLL**	0.33	6.95	[-14.68, 15.34]	0.05	13	0.962
		**RMLr**	0.17	6.95	[-14.84, 15.18]	0.02	13	0.981
**0**		**(Intercept)**	67.5	5.48	[55.66, 79.34]	12.32	13	< 0.001
	**THI**	**RLL**	3.67	5.51	[-8.23, 15.56]	0.67	13	0.517
		**RMLr**	-0.83	5.51	[-12.73, 11.06]	-0.15	13	0.882
		**(Intercept)**	40.02	7.01	[24.87, 55.18]	5.71	13	< 0.001
	**DeHb**	**RLL**	3.92	4.97	[-6.81, 14.65]	0.79	13	0.444
		**RMLr**	2.38	4.97	[-8.35, 13.11]	0.48	13	0.64
		**(Intercept)**	50.4	2.47	[44.90, 55.90]	20.42	10	< 0.001
	**StO2%**	**RLL**	-31	2.71	[-37.05, -24.95]	-11.42	10	**< 0.001**
		**RMLr**	-24	2.71	[-30.05, -17.95]	-8.84	10	**< 0.001**
		**(Intercept)**	51	6.94	[35.55, 66.45]	7.35	10	< 0.001
	**NIR**	**RLL**	-22.4	8.3	[-40.90, -3.90]	-2.7	10	**0.022**
		**RMLr**	-12.2	8.3	[-30.70, 6.30]	-1.47	10	0.173
**1**		**(Intercept)**	56.8	5.44	[44.69, 68.91]	10.45	10	< 0.001
	**THI**	**RLL**	11.8	2.36	[6.53, 17.07]	4.99	10	**< 0.001**
		**RMLr**	2.4	2.36	[-2.87, 7.67]	1.02	10	0.334
		**(Intercept)**	28.1	3.9	[19.40, 36.79]	7.2	10	< 0.001
	**DeHb**	**RLL**	27.09	3.07	[20.24, 33.94]	8.81	10	**< 0.001**
		**RMLr**	15.44	3.07	[8.59, 22.29]	5.02	10	**< 0.001**
		**(Intercept)**	47.67	5.71	[35.32, 60.01]	8.34	13	< 0.001
	**StO2%**	**RLL**	-27.33	5.14	[-38.44, -16.22]	-5.32	13	**< 0.001**
		**RMLr**	-18.5	5.14	[-29.61, -7.39]	-3.6	13	**0.003**
		**(Intercept)**	42.67	10.2	[20.63, 64.71]	4.18	13	0.001
	**NIR**	**RLL**	-10.5	8.4	[-28.65, 7.65]	-1.25	13	0.233
		**RMLr**	-1.67	8.4	[-19.82, 16.48]	-0.2	13	0.846
**2**		**(Intercept)**	50	4.73	[39.77, 60.23]	10.56	13	< 0.001
	**THI**	**RLL**	13.5	4.5	[3.78, 23.22]	3	13	**0.01**
		**RMLr**	2.83	4.5	[-6.89, 12.56]	0.63	13	0.54
		**(Intercept)**	26.43	4.58	[16.53, 36.33]	5.77	13	< 0.001
	**DeHb**	**RLL**	24.44	2.44	[19.16, 29.71]	10.01	13	**< 0.001**
		**RMLr**	10.64	2.44	[5.37, 15.92]	4.36	13	**< 0.001**
		**(Intercept)**	59	6.21	[45.16, 72.84]	9.5	10	< 0.001
	**StO2%**	**RLL**	-34.4	5.91	[-47.56, -21.24]	-5.82	10	**< 0.001**
		**RMLr**	-25.4	5.91	[-38.56, -12.24]	-4.3	10	**0.002**
**3**		**(Intercept)**	51.8	7.98	[34.01, 69.59]	6.49	10	< 0.001
	**NIR**	**RLL**	-14.4	8.85	[-34.11, 5.31]	-1.63	10	0.135
		**RMLr**	-2.8	8.85	[-22.51, 16.91]	-0.32	10	0.758
	**THI**	**(Intercept)**	50.4	5.11	[39.01, 61.79]	9.86	10	< 0.001

		**RLL**	13.2	4.27	[3.69, 22.71]	3.09	10	**0.011**
		**RMLr**	6.4	4.27	[-3.11, 15.91]	1.5	10	0.165
		**(Intercept)**	21.01	5.86	[7.95, 34.07]	3.58	10	0.005
	**DeHb**	**RLL**	27.72	4.56	[17.55, 37.88]	6.08	10	**< 0.001**
		**RMLr**	17.1	4.56	[6.94, 27.26]	3.75	10	**0.004**
		**(Intercept)**	55.5	7.07	[38.77, 72.23]	7.84	7	< 0.001
	**StO2%**	**RLL**	-7.5	5.49	[-20.48, 5.48]	-1.37	7	0.214
		**RMLr**	-2	5.49	[-14.98, 10.98]	-0.36	7	0.726
		**(Intercept)**	61	4.4	[50.60, 71.40]	13.86	7	< 0.001
	**NIR**	**RLL**	-2	3.15	[-9.46, 5.46]	-0.63	7	0.546
**4**		**RMLr**	3.5	3.15	[-3.96, 10.96]	1.11	7	0.304
		**(Intercept)**	52.75	5.47	[39.82, 65.68]	9.65	7	< 0.001
	**THI**	**RLL**	10	4.86	[-1.49, 21.49]	2.06	7	0.079
		**RMLr**	9	4.86	[-2.49, 20.49]	1.85	7	0.106
		**(Intercept)**	24.37	6.29	[9.49, 39.25]	3.87	7	0.006
	**DeHb**	**RLL**	9.3	4.33	[-0.93, 19.53]	2.15	7	0.069
		**RMLr**	4.97	4.33	[-5.26, 15.20]	1.15	7	0.289

RANDOM EFFECTS:

T	index		Parameter	Coefficient
0	StO2%	SD	(Intercept: ID)	17.87
		SD	(Residual)	7.17
	NIR	SD	(Intercept: ID)	7.59
		SD	(Residual)	12.03
	THI	SD	(Intercept: ID)	9.44
		SD	(Residual)	9.54
	DeHb	SD	(Intercept: ID)	14.87
		SD	(Residual)	8.6
1	StO2%	SD	(Intercept: ID)	3.47
		SD	(Residual)	4.29
	NIR	SD	(Intercept: ID)	8.25
		SD	(Residual)	13.13
	THI	SD	(Intercept: ID)	11.57
		SD	(Residual)	3.74
	DeHb	SD	(Intercept: ID)	7.25
		SD	(Residual)	4.86
2	StO2%	SD	(Intercept: ID)	10.79
		SD	(Residual)	8.91
	NIR	SD	(Intercept: ID)	20.31
		SD	(Residual)	14.55
	THI	SD	(Intercept: ID)	8.58
		SD	(Residual)	7.8
	DeHb	SD	(Intercept: ID)	10.4
		SD	(Residual)	4.23
3	StO2%	SD	(Intercept: ID)	10.28
		SD	(Residual)	9.34
	NIR	SD	(Intercept: ID)	11.09
		SD	(Residual)	13.99

	THI	SD	(Intercept: ID)	9.23
		SD	(Residual)	6.75
	DeHb	SD	(Intercept: ID)	10.94
		SD	(Residual)	7.21
	StO2%	SD	(Intercept: ID)	11.83
		SD	(Residual)	7.76
	NIR	SD	(Intercept: ID)	7.59
		SD	(Residual)	4.46
4	THI	SD	(Intercept: ID)	8.51
		SD	(Residual)	6.87
	DeHb	SD	(Intercept: ID)	11
		SD	(Residual)	6.12
